# Food anticipation in Bmal1-/- and AAV-Bmal1 rescued mice: a reply to Fuller et al

**DOI:** 10.1186/1740-3391-7-11

**Published:** 2009-08-10

**Authors:** Ralph E Mistlberger, Ruud M Buijs, Etienne Challet, Carolina Escobar, Glenn J Landry, Andries Kalsbeek, Paul Pevet, Shigenobu Shibata

**Affiliations:** 1 Department of Psychology, Simon Fraser University, Burnaby, BC Canada; 2 Instituto de Investigacíones Biomedicas, Universidad Nacional Autónoma de México, Mexico; 3 Institut de Neurosciences Cellulaires et Intégratives, UPR3212, Centre National de la Recherche Scientifique, Université de Strasbourg, Strasbourg, France; 4 Departamento de Anatomía, Fac de Medicina, Universidad Nacional Autónoma de México, Mexico; 5 Netherlands Institute for Neuroscience, Amsterdam, the Netherlands; 6 Department of Pharmacology, School of Science and Engineering, Waseda University, Tokyo, Japan

## Abstract

Evidence that circadian food-anticipatory activity and temperature rhythms are absent in *Bmal1 *knockout mice and rescued by restoration of *Bmal1 *expression selectively in the dorsomedial hypothalamus was published in 2008 by Fuller et al and critiqued in 2009 by Mistlberger et al. Fuller et al have responded to the critique with new information. Here we update our critique in the light of this new information. We also identify and correct factual and conceptual errors in the Fuller et al response. We conclude that the original results of Fuller et al remain inconclusive and fail to clarify the role of *Bmal1 *or the dorsomedial hypothalamus in the generation of food-entrainable rhythms in mice.

## 

Fuller et al [1] reported that *Bmal1 *deficient mice fail to anticipate a daily mealtime, and that restoration of *Bmal1 *by adenoassociated viral vector restricted to the dorsomedial hypothalamus (DMH) rescues food-anticipatory circadian rhythms. The authors concluded that the clock gene *Bmal1 *and the DMH are both required for this circadian function. However, two other labs have found that *Bmal1 *null mice do anticipate a daily meal under the same conditions as those used by Fuller et al [2,3]. Furthermore, careful scrutiny of the Fuller et al [1] article revealed numerous serious flaws in the evidence as presented in that paper, which led us to assess the results as inconclusive with respect to the role of the DMH and *Bmal1 *in the entrainment of circadian anticipatory rhythms by food [4]. Fuller et al [5] have now responded to our critique, and argue that *'each of these points *[is]*incorrect'*. Our objectives in this reply are to briefly revisit each point in the critique and to update our analysis in view of what little new information Fuller et al have provided. We also identify and correct factual errors and misconceptions in the Fuller et al response. Fuller et al conclude that '*in no case does [the new information] change our results or their import'*. We agree: the results of Fuller et al [1] remain inconclusive. Our original critique of Fuller et al contained 4 sections, addressing: 1. Data management issues, 2. Adequacy of data to support the major claims, 3. Missing data or methods, and 4. Conceptual issues. We now re-examine each point.

**1a**. The original Fuller et al [1] article contained a duplicate figure, one version intended to illustrate that viral-mediated restoration of *Bmal1 *expression in the DMH rescues food anticipatory rhythms, and the other version of the same data intended to illustrate that restoration limited to the SCN does *not *rescue food anticipatory rhythms (see Fig. 1 in [4]). The second version was misaligned (time-shifted by 3-h), contained a 3-h gap deleting evidence for anticipation, and misidentified the feeding time. The duplicate was later replaced on-line with a Correction notice (*Science*, Oct 31, 2008). Given that the Fuller et al [1] study included only 4 mice in the *Bmal1*-DMH rescue group, a mistake of this magnitude in such a small dataset raises serious concerns about data management.

**Figure 1:**
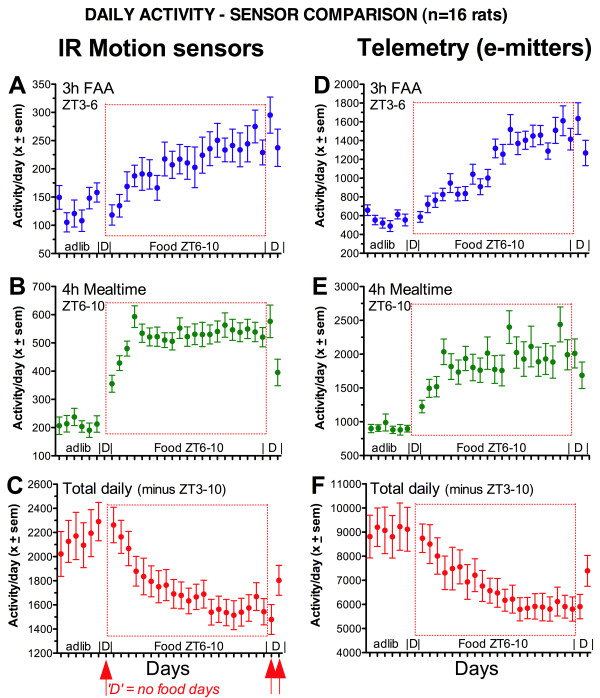
**Mean daily activity counts detected by infrared (IR) motion sensors and by intraperitoneal transponders (e-mitters) in the same adult male rats (n = 11 DMH sham lesions and n = 5 unoperated controls, combined; data from Mistlberger et al **[12]). The motion sensors were positioned above the middle of the cage, as in [12,15]. A, D. Mean counts during hours 3–5 of the light period, corresponding to the time that food-anticipatory activity appears when food is restricted to hours 6–10. B, E. Activity during hours 6–10 of the light period, corresponding to mealtime during restricted feeding. C, F. Total daily activity excluding hours 3–10 of lights on (3 h premeal and 4 h mealtime). Abbreviations: Adlib denotes baseline days with food available ad-libitum. 'D' denotes days when food was not provided (also indicated by arrows below panel C). ZT denotes 'zeitgeber time', referring to the number of hours after lights-on each day (ZT0 by convention). Days when food was restricted to ZT6–10 are further identified by the dotted line box in each panel.

**1b**. The 4-day average waveforms of core body temperature (T_b_) in Figs. 2A, 2C, and S 3C of Fuller et al [1] are highly anomalous, as they indicate that T_b _in these mice peaked at or in the hour before mealtime, and then dropped precipitously by up to 3°C while the mice ate. These patterns are anomalous because feeding increases T_b _in rats and mice. These patterns are consistent with errors in data alignment, i.e., data time-shifted to the left. Fuller et al reply that '*those are the data'*, and cite two other studies as ostensibly showing the same result [6,7]. However, the waveforms in these other studies are nothing like those in Fuller et al. In both studies, T_b _in mice was shown to rise during the first ~30 min of feeding, and either stay high or gradually decline over 4 h back to the level exhibited just prior to meal onset, not 3°C below as in Fuller et al [1]. The reader is referred to these and other mouse food restriction studies [6-11]. Fuller et al [5] fail to acknowledge these obvious differences or to provide an explanation for why some of their mice *do *show a marked thermogenic effect of eating (e.g., Figs. 2B, S 2D in [1]), while other mice exhibit patterns without precedence in the literature.

**1c**. Fuller et al [1] show T_b _data from two individual mice in both raster format (*'actigram'*, a misnomer, given that these are temperature, not activity data) and as 4-day average waveforms. The two versions of each data set exhibit discrepancies in the timing of peaks and troughs. Fuller et al [5] reply that the plots are correct. However, if one plotting format shows high or low temperature where another format does not, there is something wrong with either the algorithms or data alignment.

**1d**. Figs. S 3A and S 3B in Fuller et al [1] are identified as food restricted mice in constant dark (DD), yet in both cases the activity patterns look remarkably like wildtype mice entrained to LD, with feeding time in the mid-light period. These rhythms are highly anomalous compared to the rhythms of food-restricted mice housed in DD in other published studies (e.g., [7,8]). Fuller et al [5] respond that the mice are in fact in DD, but fail to acknowledge how anomalous these figures look.

**2a**. Fuller et al [1] claim to have restored *Bmal1 *expression selectively in the SCN or DMH by locally injected viral vector. In their original paper they provided only cropped images of the SCN or DMH, acceptable for many studies, but not adequate evidence in a study claiming spatial selectivity of rescued gene expression. Fuller et al [5] now provide a full series of *Bmal1 *autoradiographs from mice with *Bmal1 *expression restored selectively in the SCN or DMH. They do not tell us whether these were from mice in the original study, and if so, to which temperature plots they correspond.

**2b**. Fuller et al [1] claim to have restored food anticipatory temperature rhythms by rescuing *Bmal1 *expression in the DMH, but the T_b _plots in their original paper appeared to be cropped to include only the food restriction days, and no baseline days (ad-lib food access) to demonstrate that rhythms apparent during food restriction were not already present during ad-lib food access. Fuller et al [5] now clarify that the first day in these charts was actually the baseline day, and that the red line through that day indicating scheduled mealtime was another mistake in the original paper. Therefore, the charts were not cropped, but were mislabeled, and the study was designed to include only a single day of baseline, which is inadequate to assess the presence or absence of rhythms prior to restricted feeding. To conduct such a challenging gene rescue experiment, but include only a single baseline day in the food restriction experiment is a remarkable oversight, especially given that Fuller et al [1] collected several weeks of LD and DD data to show that rescue of *Bmal1 *in the SCN restored both LD entrainment and free-running circadian rhythms (confirming what is already well-established, that the SCN is a *Bmal1*-dependent circadian pacemaker).

**2c**. Fuller et al [1] claim that food anticipatory rhythms in DMH-*Bmal1 *rescued mice persist during a 24 h food deprivation test, thereby '*demonstrating the circadian nature*' of these rhythms. However, a 24 h food deprivation test proves nothing, as it is no different from a regular day of food restriction. It is the fall and then rise of temperature and activity at the expected feeding time on the next cycle after a skipped meal that demonstrates the operation of a self-sustaining circadian oscillator, and that rules out a metabolic hourglass timer. In their reply, Fuller et al '*disagree' *that a 24 h food deprivation is an inadequate test, and state that *'if the anticipatory rhythm were an hourglass or interval timer effect, it would continue through the presumptive feeding period, as the animal became more and more hungry. If the rhythm represented circadian entrainment, it would collapse at the time of the presumptive food presentation, even though no food had been given'*. This argument is neither theory- nor evidence-based. First, circadian entrainment theory does not predict an immediate 'collapse' of the rhythm if food doesn't appear at the usual meal onset time. Second, decades of empirical evidence show that in intact rats and mice, the rhythm does not 'collapse' at the time of food presentation if no food is provided. In limited-duration food restriction studies (in which food is provided for a fixed amount of time/day), activity and T_b _remain elevated until the normal duration of food availability is past, and only then do they decline (e.g., [4,7,12]). Fuller et al's [5] claim that T_b _and activity 'collapse' at the beginning of the expected feeding time on meal omission test days is peculiar to their data.

**2d**. Fuller et al [1] claim that *Bmal1 *null mice do not show a rise in T_b _prior to mealtime, but regression lines fit to the T_b _waveforms provided in their paper clearly indicate that T_b _was increasing prior to mealtime (see Fig. 8 in [4]).

**2e**. Fuller et al [1] reported that *Bmal1 *null mice exhibited torpor during food restriction, and had to be woken up at mealtime '*to prevent their starvation and death'*. Two other groups that have also conducted food restriction experiments on *Bmal1 *mice were careful to introduce food restriction gradually, observed robust food anticipatory behavior, and never had to physically arouse the mice for them to feed, because they were already awake and actively anticipating mealtime [2,3,13]. It bears repeating: A hungry mouse that does not arouse on its own when an isolation box is opened and food is placed on or in a cage is either torpid or very sick, due to malnutrition. Fuller et al [5] now provide body weight curves indicating minimal body weight loss in their mice, although their original paper [1] provides no indication that the mice were weighed during the experiment. Two other groups report that the food restriction procedure described by Fuller et al [1] results in rapid weight loss in mice without running wheels (J. Pendergast, S. Yamazaki and W. Nakamura, data in [4]), with high mortality rates in mice with wheels [3].

**3a, 3b**. The Fuller et al [5] response now provides the number and age of the mice used in their study.

**3c**. The response [5] indicates that a technician did the viral injections and provides approximate hit rates for injections and success rates for *Bmal1 *rescue.

**3d**. The response [5] indicates that no mice in these groups were excluded from Fig. 2D in Fuller et al [1]. A new figure is provided (Fig. 2 in [5]), showing T_b _during the 4 days used to generate Fig. 2D in [1], from two of the mice. The blue curve (a *Bmal1+/- *mouse, in which the circadian clock is functional) is strikingly anomalous by comparison with the results of other studies of T_b _rhythms in food-restricted mice. On at least 3 of the 5 days illustrated, T_b _exhibits an almost square wave, near 2°C step increase in the hour or so prior to mealtime. This pattern is without precedence in the literature. Fuller et al [5] fail to comment.

**3e**. Fuller et al [5] state that hypothermia occurs at night in food restricted mice fed during the day, and cite several studies. These studies show that temperature declines through the night, but that the timing of the nadir depends on feeding time; when meals are in the midday, the nadir occurs during the first hours of the light period (e.g., [6,7]).

**3f**. Fuller et al [5] indicate that the discrepancies in cage temperature reported in the original Supplementary Materials (24°C) and in the published Correction (22°C) were due to poor communication between co-authors, and failure of one author to check revisions made by another. Fuller et al [5] state that the thermoneutral zone is 29°C in mice. However, the thermoneutral zone is a range of temperatures, not a point, and it varies by species and strain, and may differ in *Bmal1 *null mice, which have metabolic defects.

**4a**. Fuller et al [5] in this section repeat an argument made earlier in their response that neural and molecular mechanisms of food anticipatory rhythms should only be studied using behavioral or physiological endpoints that are unchanged or are decreased in mean daily level by food restriction. They reason that if food restriction stimulates a behavior, such as wheel running, level pressing or food bin approaches, then it is impossible to know whether a premeal increase in the behavior is due to circadian timing or due to a homeostatic hourglass process (activity stimulated by hunger). However, this argument is spurious [14]. First, when rats are food deprived for up to 3 days, they do not exhibit a mid-day peak of running or general activity unless they have been previously fed at that time of day (e.g., [15-17]). Second, whether the mean daily level of a behavior increases, decreases or stays the same during food restriction reveals nothing about the mechanism underlying expression of that behavior prior to mealtime. It is irrelevant. The mechanism can only be classified by tests to characterize formal properties of the rhythm. The simplest test is to skip one or more meals. If the anticipatory behavior remains concentrated at the expected feeding time the day after a scheduled feeding is skipped, then this behavior cannot be accounted for by an hourglass process, regardless of whether its mean level is higher, lower or unchanged during the challenge. In mice (unlike rats), food deprivation tests can be problematic, because hyperactivity or hypoactivity (and hypothermia) may rapidly ensue if a meal is skipped, which may obscure behavioral output of a food-entrainable clock. In that case other tests are required, such as meal shifts and T-cycle experiments, to determine whether the food anticipation rhythm behaves like the output of an entrained oscillator or like an hourglass timer [17-19].

Fuller et al [5] maintain that activity measured by telemetry (the only behavioral variable assessed in their studies) does not increase during food restriction, while activity measured by overhead motion sensors does increase, and that therefore the former is a pure measure of circadian regulation while the latter is confounded by 'homeostatic' factors. To evaluate this unsubstantiated claim, we measured activity by telemetry and infrared (IR) motion sensors in the same rats, and showed that the two measures produce virtually identical patterns under ad-lib and restricted feeding conditions [12]. The make, model and position of the IR motion sensors was the same as in the Landry et al study [15] that showed normal food anticipatory activity in rats with complete DMH ablation. It is therefore reasonable to infer that telemetry would also have revealed normal food anticipation in those rats. The same results have been obtained in sham and DMH-ablated mice, using the same sensors (Sellix and Menaker, personal communication, July, 2009).

Fuller et al [5] in their response claim that normalization of the data (as presented in [12]) invalidates the comparison between IR sensors and telemetry. On the contrary, normalization is the correct procedure for comparing the timing of two variables that have different mean levels (telemetry produced ~4 times more counts/day than the motion sensors, which have a refractory period of 10 sec after each trigger). Fuller et al [5] call our use of normalization [12] a '*deliberately deceptive' *attempt to hide a change in mean levels, and state that '*one measure *[is]*substantially increased by food restriction and the other is dramatically decreased'*. This statement is again not evidence-based. We 'deliberately' did not report absolute values in that paper because it is irrelevant, but provide them here (Fig. 1). When food was limited to hours 6–10 of the light period for 18 days, both telemetry and IR motion sensors generated fewer activity counts per day by comparison with baseline ad-lib food access, and the magnitude of this change did not differ between measures (-16.5 +- 10.8% and -12.6 +- 14.0%, respectively, using grand means from 5 baseline and 18 food restriction days in 16 rats; paired T_(15) _= 1.31, p = .21). During a subsequent 50 h food deprivation test, telemetry and motion sensor counts increased but remained below baseline levels (7.1 +- 12.8% and 6.2 +- 15.9% lower, respectively).

It bears emphasizing, the criterion for selecting a measure of food anticipatory activity (or physiology) should not be whether mean daily levels of this measure go up or down during food restriction. Rats and mice reduce total daily energy expenditure when calories are restricted, and this will be reflected in reduced activity at times of day when food is not expected. They are able to do this by utilizing a circadian clock to concentrate food seeking at the expected mealtime. General measures of activity, such as provided by telemetry or IR motion sensors, are adequate to detect food anticipation in neurologically intact, wildtype rats and mice, but such measures sometimes fail to detect circadian clock-controlled food anticipation revealed by foraging-related measures (e.g., [20]). The use of telemetry alone to measure general activity in rats or mice living in barren cages is sufficient if food anticipation is evident and circadian properties have been tested appropriately; it is *not *sufficient if food anticipation is weak or absent. Food-anticipation is clock-controlled foraging, and more sophisticated behavioral assays (feeding locations, operant behaviors) should be employed to properly phenotype this circadian function.

**4b**. Fuller et al [5] state that Mistlberger et al "*consistently misrepresent what we *[1,21]*have shown and how we frame it*". This is a puzzling complaint. To remind the reader, the Saper laboratory [21] first reported that partial DMH lesions attenuate or eliminate food anticipatory rhythms in rats. They concluded from this that the DMH is '*critical' *for food entrainment. That is in the title of their paper. Fuller et al [1] then claimed that rescuing *Bmal1 *expression in the DMH is sufficient to restore food anticipatory rhythms. Taken together, the two studies claim to have demonstrated that the DMH is both necessary and sufficient for food entrainment, and that his how we have summarized their findings. Fuller et al [5] say that '*This is not correct'*. Yet, in the final paragraph of their response (also summarized in the Abstract), they state that '*we stand by our findings that the DMH is necessary for organizing food entrained circadian rhythms, and that ...robust activation of ... clock genes in the DMH is sufficient to restore food-entrained rhythms'*. There are many other food-entrainable circadian oscillators in brain and body, but if the DMH meet criteria for necessity and sufficiency, as claimed, then these other oscillators cannot also be both necessary and sufficient for food anticipatory activity rhythms.

Fuller et al [5] state that the Saper group's [21] DMH lesion study shows only that '*the food entrainable oscillator uses the same output mechanisms through the DMH as does the SCN'*. However, lesion data cannot establish whether the DMH is input, oscillator or output, only whether it is or is not '*critical'*.

## Other issues

### Classical conditioning and hourglass processes

Fuller et al [5] argue that, to interpret the presence of food anticipatory rhythms in animals with brain lesions or gene mutations, it is important to rule out external cues and internal homeostatic factors that might compensate for loss of a food-entrainable circadian timer. This is not news; for thorough discussion, see [17] and a forthcoming special issue of the *European Journal of Neuroscience *[22-26]. Fuller et al [5], however, stumble in their effort to apply these concepts. Fuller et al state that rats or mice lacking a food-entrainable clock but tested in a LD cycle might '*learn cognitively' *by '*classical conditioning' *that food is delivered each day in the middle of the light period (either 5 or 6 h after lights-on). They attempt to support this argument by stating that in the Moriya et al DMH lesion study (Fig. 8C in [7]), food anticipatory activity '*in two mice' *was reduced by '*about 25%*' during two test days in DD compared to the previous days in LD. Neither the theoretical formulation nor the representation of data from Moriya et al [7] are correct.

First, for classical conditioning to occur, the conditioned stimulus (CS, light) and the unconditioned stimulus (US, food) need to be closely spaced in time if not contiguous. Classical conditioning does *not *generate a conditioned response (CR, food anticipation) if there is a 5–6 h gap between the CS onset and the US (excepting the special case of conditioned food avoidance). Furthermore, if the CS (light) is omitted, then the CR (anticipation) should be absent. If food anticipatory activity were a CR to an association between light and feeding, then it would begin at light onset and would be completely absent in DD, not attenuated 25%. Food anticipation in Moriya et al [7] exhibited neither property.

Second, as is clearly stated in the figure legend, the waveforms in Fig. 8C in Moriya et al [7] illustrate group means, not individual mice. Each data point is expressed as a percentage of total daily activity, so it is meaningless to compare the DD day to the LD day in this way, as if the percentages represented absolute counts. The amount of anticipatory activity in each time bin as a percent of total daily activity will depend on how much activity there is at other times of day (food was omitted on the DD day, therefore activity was sustained at a higher level throughout the mealtime, as expected, and activity prior to mealtime represented a smaller percent of total daily activity). The point of this figure is to illustrate that the timing and magnitude of food anticipatory activity in DD are virtually indistinguishable in DMH lesion mice compared to sham lesion mice. That is the critical issue: do mice with DMH lesions anticipate mealtime or not? They do, whether in LD or DD. Percentages are used to normalize the data, so that both groups can be plotted on the same axis despite lower mean daily counts in the lesion group.

### Lesion validation and methods

Fuller et al state that the 'electrolytic' (*sic*, radiofrequency) DMH lesions in Landry et al [15,27] were not validated, because the study did not assess physiological and behavioral markers that they claim are indicative of complete DMH lesions. However, the Saper laboratory has not reported on complete DMH lesions, because the neurotoxin used in their study [21] produces only partial lesions. Also, the Saper laboratory have used only telemetry to measure activity, and thus cannot make claims about how other measures are affected by DMH lesions. Validation of lesions requires visual inspection of post-mortem brain tissue. Behavior and physiology provide lesion correlates, not validation. The statement of Fuller et al [5] that electrolytic lesions distort the brain to the extent that lesion validation is impossible is false. The lesions in Landry et al [15,27] were described and illustrated in detail, and the reader is referred to the figures in those manuscripts. Despite the large cavities where the DMH would normally reside, the brains are *not *distorted, and major landmarks (fiber tracts and other nuclei) are clearly evident, permiting an accurate assessment of what structures are damaged or absent. Electrolytic and radiofrequency lesions are the only way to unambiguously remove the entire DMH. The neurotoxin used by the Saper group makes only partial lesions, and requires cell counting to estimate lesion size. Cell counting sounds rigorous but it is problematic if the cells are not phenotyped and the boundaries between areas are normally unclear or have become obscured by loss of cells. The rational approach to assessing whether a structure is necessary for a particular circadian behavior is to ablate the entire structure and surrounding area. If there is a loss of function, then cell-specific lesion techniques can be used to assess a role for fibers of passage or specific cell phenotypes. We and others find no loss of food anticipatory activity rhythms despite complete removal of the DMH and fibers of passage ([7,15,27], Sellix and Menaker, personal communication, 2009). The apparently different behavioral effects of complete DMH removal by tissue ablation versus partial DMH lesion by neurotoxin remain to be explained, if the neurotoxin results stand up to replication.

### The role of clock genes and the DMH

Although the Saper laboratory has claimed that the DMH is critical for the expression of food anticipatory activity rhythms [21], others have shown that food anticipatory activity does not require the DMH, and one or more of these studies have ruled out LD cues and metabolic hourglass processes as compensatory mechanisms ([7,15,27], Sellix and Menaker, personal communication, 2009). We therefore conclude that the DMH is not necessary for food-entrainable behavioral rhythms. The DMH may play a modulatory role in the expression of food anticipatory rhythms (e.g., [28,29]), or it may participate as part of a larger population of oscillators in the mediobasal hypothalamus [30] that might together drive anticipatory rhythms. Such roles have not been ruled out. Although we reject the conclusions of Fuller et al [1], due to its critical methodological flaws, we do not rule out a role for *Bmal1 *or other clock genes (e.g., [8]) in the operation of food-entrainable clocks. Two other labs have shown that *Bmal1 *null mice exhibit clear food-anticipatory activity in LD or DD [2,3], directly contradicting the claims of Fuller et al [1]. However, no study has shown that food anticipatory rhythms clearly persist during total food deprivation (meal omission) tests in *Bmal1 *knockout or rescued mice; neither Fuller et al [1] nor Storch and Weitz [3] employed food deprivation tests of the required duration (due to university-specific regulations), while Pendergast et al [2] observed weak or no clear expression of activity at the prior feeding time during food deprivation tests. Food-entrainable rhythms may lose self-sustaining properties in *Bmal1 *deficient mice, or this property may be obscured by metabolic deficiencies in knockout mice (causing hyperactivity or hypoactivity, which mask clock output). As noted already [18,19,23,25,26], additional tests of food-entrained circadian properties in clock gene knockout and wildtype mice are needed (e.g., entrainment limits and meal shifts).

### Collegiality

Two months prior to writing the critique [4] of Fuller et al [1], Mistlberger and another colleague (Dr. Alec Davidson, Morehouse School of Medicine, GA) communicated with Dr. Fuller via email, seeking an explanation for problematic features of the duplicate temperature plots published in Fuller et al [1]. Dr. Fuller provided missing information on the plotting conventions, which confirmed that the duplicate plots should not be identical if one version contained a true data gap. However, Dr. Fuller did not contest or correct our statement that the plots indeed appeared to be identical. Dr. Fuller also did not respond to our eventual request to share raw data files. Nonetheless, the failure to obtain an adequate explanation for one figure did not motivate writing of the critical review [4]. Rather, the review was undertaken to provide an expert, consensus re-evaluation of what can and cannot be concluded from the entire set of results in that high profile publication. Critiques of specific papers are common in the literature, and serve an essential function (see 'Technical Comments' in *Science *for many examples). Our critique is unusual only in its length. The response by Fuller et al [5] only serves to validate our analysis.

## Competing interests

The authors declare that they have no competing interests.

## Authors' contributions

The manuscript was drafted by RM and the final version read, edited and approved by RB, EC, CE, GL, AK, PP and SS.
